# Automated pencil electrode formation platform to realize uniform and reproducible graphite electrodes on paper for microfluidic fuel cells

**DOI:** 10.1038/s41598-020-68579-x

**Published:** 2020-07-15

**Authors:** Lanka Tata Rao, Prakash Rewatkar, Satish Kumar Dubey, Arshad Javed, Sanket Goel

**Affiliations:** 1 Department of Mechanical Engineering, Birla Institute of Technology and Science (BITS) Pilani, Hyderabad Campus, Hyderabad, 500078 India; 2 MEMS, Microfluidics and Nanoelectronics Lab, Department of Electrical and Electronics Engineering, Birla Institute of Technology and Science (BITS) Pilani, Hyderabad Campus, Hyderabad, 500078 India

**Keywords:** Electrical and electronic engineering, Mechanical engineering, Materials for devices, Materials for energy and catalysis, Techniques and instrumentation

## Abstract

Graphite pencil stroked electrodes for paper-based Microfluidic devices are gaining immense attention due to their electrochemical properties, cost efficiency, and ease-of-use. However, their widespread use has been hindered by the challenges associated with their manual fabrication such as non-uniformity in graphite deposition, applied pressure, etc. This work presents the design and development of an automated graphite pencil stroking device for graphite electrode fabrication with high efficiency through a compact, inexpensive and automatic process, with reduced fabrication time and human intervention leading to more uniformity. The motion platform of Graphtec plotter was used to create multiple strokes with the help of the proposed device. Such inexpensive graphite electrodes (less than the US $1) have been observed to be porous in nature, acting as diffusion agents. The automated graphite electrodes were used to study the performance of microfluidic paper fuel cells (MPFCs) with formic acid, oxygen, and sulphuric acid acting as fuel, oxidising agent and electrolyte respectively. From this configuration, the maximum current density and power density were measured to be 1,305.5 µA cm^−2^ and 135.5 µW cm^−2^, respectively at 0.3 V stable OCP at 100 strokes. Overall, the study enumerates the development of an automated pencil stroke device for fabricating graphite electrodes, which can potentially be harnessed in numerous miniaturized paper based applications.

## Introduction

Microfluidic devices with integrated electrodes, or electromicrofluidic devices, have attained essential roles in diverse areas, including energy harvesting for portable applications and sensing devices^1–3^. Paper–pencil based microfluidic fuel cell is one of the most recent vital advancements to develop point of the source (POS) and point of care (POC) devices owing to the well-proven benefits of microfluidic environment and graphite electrodes^4,5^. In recent years, graphite pencils are being used as electrodes and have shown encouraging outcomes and promising features of MPFCs when compared with the existing approaches. Initially approaches on graphite electrodes, Aoki et al. demonstrated the first use for of graphite material (pencil lead), as electrodes^6^. Bandapati et al. investigated the performance of membraneless glucose biofuel cell with various grades of graphite pencils act as electrodes^7,8^. Rewatkar et al. examined 3D printed enzymatic biofuel cell performance with graphite pencil as an electrodes^9^.

In spite of the numerable benefits of such devices, they come with an inherent setback of the physically fabricated electrodes with non-uniform deposition of graphite due to the subjective nature of the applied pressure and manual counting of the number of strokes. Most of the recent researches in the field of paper–pencil based microfuel cells employ manual deposition of graphite on the paper for fabricating the electrode. Arun et al. reported formic acid microfluidic fuel cell with manual graphite pencil electrodes with diverse grades of pencils^10,11^. Veerubhotla and Ye et al. studied performance with graphite pencil stroke electrodes on paper based microbial fuel cells^12,13^. Lal et al. showed a paper based microfluidic fuel cell using different filter papers, whereby graphite sheets acted an electrode, and formic acid and potassium permanganate acted as a fuel and an oxidant, respectively^14^. Shen et al. studied the influence of performance on paper based fuel cells at fluidic concentration variation^15^. Further, the required electrodes can be integrated into the microchannel with sufficient distance between electrodes, from the depletion to the diffusion zones, to reduce the effect of fuel mixing^16^. In the earlier work by the authors, Rao et al. investigated pencil paper based fuel cells with formic acid acting as fuel, oxygen as an oxidant, graphite electrodes as electrodes. Such graphite electrodes were realized by manual strokes with varying numbers of strokes and with diverse grades of pencils^17,18^. Jung et al. investigated and presented a paper based vanadium co laminar micro fuel cell where electrodes were fabricated by screen printing of water dispersible graphene paste. They reported a maximum current density of 550.5 μA cm^−2^ and 445.0 μA cm^−2^ for graphene paste electrodes and manual pencil stroked electrodes fuel cell, respectively^19^. Overall, in most of the reported work, the pencil electrodes have been physically realized.

Evidently, the quantity of graphite deposited on the paper affects the performance of the fuel cell, which can be modified by varying the number of pencil strokes^18^. This manual process for electrode fabrication brings in scope for many precisions related variations and errors due to human intervention, as the designer does not have any precise control over the amount of graphite deposited. Further, the amount and quality of the graphite being deposited and adhered on the surface can vary from person to person and time to time depending upon the pressure applied. The quantity of graphite deposited on the paper affects the performance of the electromicrofluidic device and can be modified by varying the number of pencil strokes. Based on this, it can be concluded that there is a need for a simple, user-friendly, and reliable platform device for graphite electrodes formation in an automated manner.

The present work describes the design and development of an automated graphite pencil stroking device for graphite electrode fabrication with uniformity in applied pressure during electrode fabrication leading to even graphite deposition on porous cellulose paper. The proposed device addresses the aforementioned problems and provides an integrated solution for economical, easy to use, uniform and, a portable, automated device for graphite electrode on for paper based devices. Such automated graphite electrodes have excellent uniformity, high efficiency, and can be fabricated inexpensively. Moreover, the quantity of force can also be identified at the time of graphite electrode fabrication time by force sensing resistor (FSR) sensor. The platform has been harnessed to be used to realize microchannel and electrodes of microfluidic paper fuel cell (MPFC) showing excellent power output. Overall, the platform is an accurate, fully-automated device for graphite electrode fabrication on porous cellulose paper for sensing, energy harvesting, and flexible electronics applications.

## Result and discussion

### Graphite electrodes fabrication

**Figure 1:**
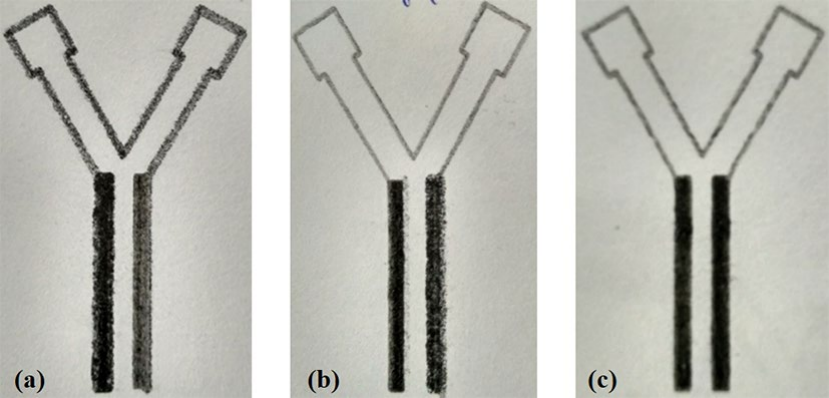
Automated graphite pencil stroke electrodes combinations with different strokes, (**a**) 8B-HB at 10 strokes, (**b**) 8B-HB at 15 strokes, and (**c**) 8B-HB at 30 strokes.

Graphite pencil electrodes were fabricated on porous natured Whatman filter paper (Gr 1) by automated pencil stroke device with Graphtec plotter for microfluidic paper fuel cells (MPFCs). As shown in Fig. 1, the rectangular graphite electrodes (3.0 × 0.2 cm) were prepared on a Whatman Gr 1 paper along the length of the microchannel. In this configuration, the active surface area of the graphite electrodes was 0.6 cm^2^. Here, two diverse grades of graphite pencils, HB and 8B, were used to prepare electrodes with various strokes, with constant force and uniform graphite particle deposition. The choice of the graphite pencils was based on the earlier published work by our group^18^.

As shown in Fig. 1, the automated pencil holder device was used to create various combinations (8B–8B, 8B–HB, HB–8B, and HB–HB) of graphite electrodes with uniform graphite deposition and equal force. A real-time video of graphite electrodes formation is presented in supplementary video S1.

**Figure 2:**
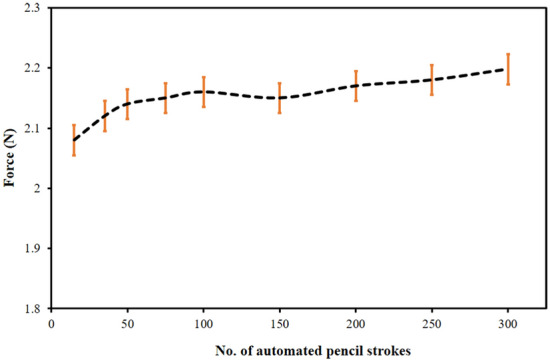
Automated graphite pencil electrode force at the time of fabrication time with a different number of strokes with a standard deviation (n = 3).

Subsequently, the force was identified by the FSR sensor at the time of the fabrication of the graphite electrodes. The step-by-step force-sensing operation of the FSR sensor is mentioned in Fig. S1. The value of force for various graphite pencil strokes with an error bar is shown in Fig. 2, whereby the standard deviation (± 0.05 N) was calculated with ‘n’ performed experiments (n = 3) showing excellent consistency in the force up to more than 300 strokes with an average force of 2.1 N.

### Automated graphite electrodes characterization

**Figure 3:**
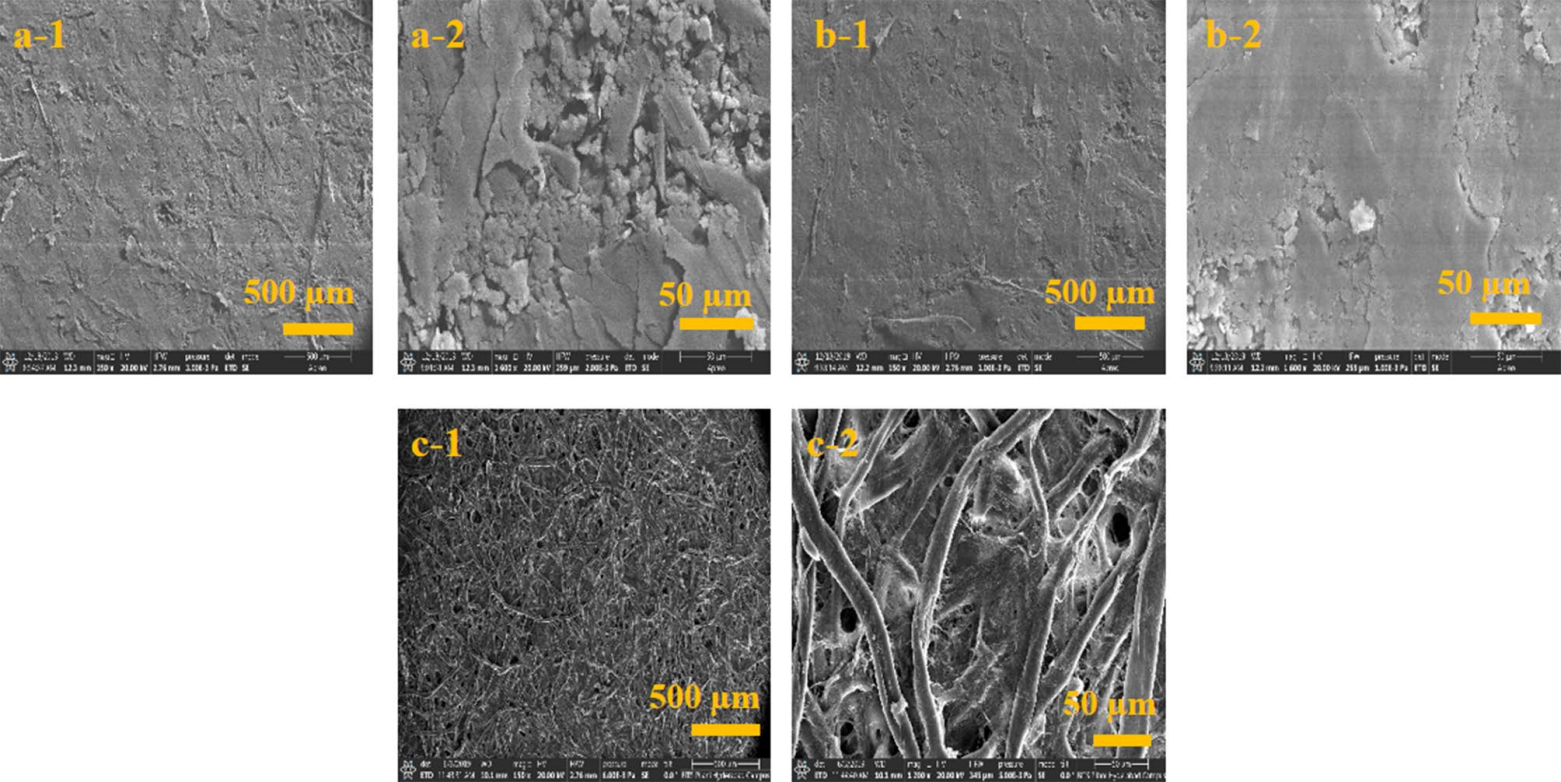
SEM images of automated and manual graphite pencil strokes on Whatman filter paper at 50 strokes, (**a**) automated pencil strokes at different scale bars (500, 50 µm), (**b**) manual pencil strokes at different scale bars (500, 50 µm), and (**c**) Whatman filter paper (microchannel) at different scale bars (500, 50 µm).

The surface morphology of automated and manual graphite pencil stroke electrodes, fabricated on porous nature Gr1 Whatman cellulose filter paper with graphite pencils (HB, 8B), at 50 strokes, were captured by scanning electron microscopy (SEM) and compared. As can be seen in Fig. 3, the enhancement, in terms of uniformity and repeatability, of graphite particle deposition with automated pencil stroke electrodes is more than the manual stroke electrodes. Figure 3 presents the microstructure (morphology) analysis of Gr 1 Whatman filter paper for automated graphite electrodes and manual stroke electrodes at different magnifications (500, 50 µm). Here, the primary aim of the SEM analysis was to identify the microstructure (pore size) of graphite electrodes in comparison with the plain filter paper (without graphite content).

In Fig. 3, automated (a-1, 2), and manual (b-1, 2) pencil strokes have been compared with the plane filter paper (c-1, 2). It can be observed that the effects of manual and automated strokes are different on the filter paper. The graphite scales formed through the automated process produce air breathable pores which utilise capillary transport and thus yield highly efficient electrodes as compared to the manual one. It can be observed that the deposition of graphite on the filter paper retains the porous property of the filter paper. The distorted graphite scale formation in the filter paper provides better air breathing capability to the electrode and thus leads to better performance.

Further, surface elemental characterization was performed by EDX analysis (energy dispersive X-ray). The difference between automated and manual composed graphite electrodes was observed, and surface elemental composition was evaluated with standard deviation. As shown in Table 1, the elemental composition of automated and manual composed graphite electrodes with 8B graphite pencil at 50 strokes was analysed. After an in-depth analysis of standard deviation, automated graphite pencil electrodes showed high uniformity, accuracy, and repeatability than manual pencil strokes, which can be interpreted from Table 1.

**Table 1:** Detail summary of EDX characterization for automated and manual pencil strokes on porous nature Whatman filter paper (Gr 1) with standard deviation (n = 3).

S. no.	Elements (K) %	Automated pencil strokes			Manual pencil strokes		
		Average value	Standard deviation	Standard error	Average value	Standard deviation	Standard error
1	C	72.087	0.148	0.086	68.22	3.36	1.94
2	O	17.157	0.408	0.236	26.813	1.992	1.15
3	Si	5.39	0.384	0.222	2.953	0.531	0.306
4	Fe	2.157	0.097	0.056	1.15	0.087	0.05
5	Al	1.497	0.441	0.255	1.08	0.165	0.095

### Application of an automated pencil stroke device fabricated electrodes

After successful graphite electrode fabrication using the proposed platform, such electrodes were utilized in a microfluidic paper fuel cell (MPFCs) with formic acid (1 M) acting as fuel, sulphuric acid (3.75 M) as an electrolyte, and oxygen as oxidant. Here, two different automated graphite electrodes (HB, 8B) were prepared with 30, 50, and 100 strokes with equal force and uniformity. From the literature, the graphite content present in graphite pencils were 68% in HB and 91% in 8B^20,21^. The force (N) was measured at the time of electrodes fabrication, and an explanation is given in “Automated pencil stroke device setup with plotter” and Fig. 7. The fabrication, assembly, and characterization of the microfluidic paper fuel cells (MPFCs) were similar to our previous work^18^. The complete experimentation setup with various automated graphite electrodes (HB, 8B) is shown in Fig. S2.

**Figure 4:**
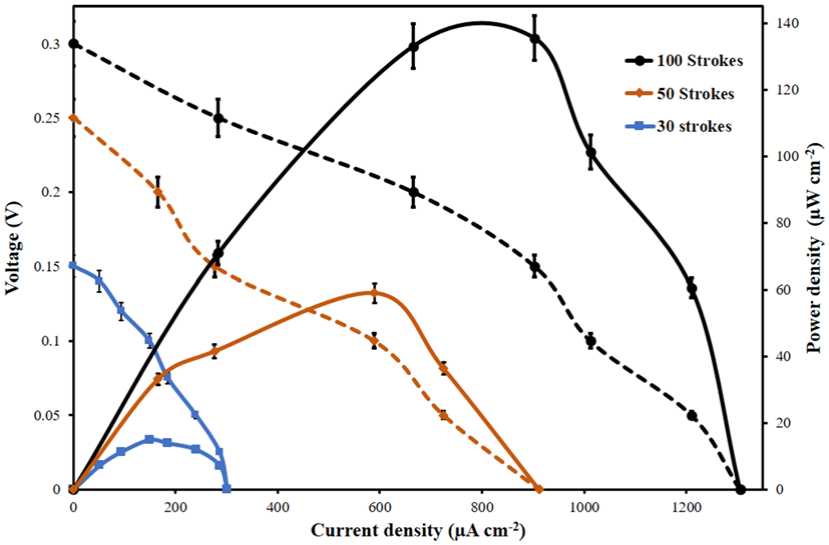
Polarization performance of microfluidic fuel cell with automated pencil electrodes at 30, 50, and 100 strokes with standard deviation (n = 3).

Initially, an Open Circuit Voltage (OCP) was observed in a MPFC with automated graphite electrodes. Subsequently, the polarization performance of MPFC was investigated by using the chronoamperometry technique with stable OCP of 300 mV. The microfluidic fuel cell polarization curves with various automated pencil strokes (i.e., 30, 50, and 100) are shown in Fig. 4.

The electrochemical characterization of MPFCs was carried out with several automated pencil electrodes with a varying number of strokes. These characterizations were carried out based on energy density (i.e., current density and power density) with the measured open circuit potential (V). In such configuration, the maximum current and power densities were observed to be 1,305.6 µA/cm^2^ and 135.504 µW/cm^2^, respectively, with 100 automated pencil strokes.

The aforementioned automated graphite pencil stroke device with plotter has shown excellent compatibility, robustness, and reproducibility. The fabrication process of the uniform electrodes is simple and inexpensive compared to the manual graphite stroke electrodes. It is clear that automated graphite pencil stroke device has a strong potential to enable various low-power applications, flexible electronic application, and sensing applications.

## Conclusions

This paper presents the design and development of an automated pencil stroke formation device for electrode fabrication. Following are the main conclusions and the prominent features of the proposed device:

- The device has a promising potential for large scale fabrication of automated electrodes with uniform deposition and constant force at the time of electrode fabrication. Such fabricated electrodes can be utilized for energy harvesting, flexible electronics, and sensing applications.
- Different grades of graphite pencils such as HB, 8B, and a varying number of strokes can be used to obtain electrodes with varying properties and efficiency on porous Whatman filter paper as per the requirement with the help of automated pencil stroke device with graphtec plotter.
- After rigorous experimentation, characterization, and analysis, MPFC with automated graphite pencil electrodes show the optimum performance with power and current densities of 1,305.6 µA/cm^2^ and 135.504 µW/cm^2^ respectively at 0.3 V stable OCP at 100 automated pencil strokes.
- The proposed device also ensures uniform application of force at the time of electrode fabrication. The fabricated automated electrode device is very inexpensive (less than the US $1) and electrodes fabricated with this device can be used in a wide variety of applications.
- The proposed device provides a platform to develop standardized electrodes, which can be used for various applications where the electrical output is needed. These applications include microfluidic fuel cells, electrochemical and amperometric sensors for toxic and nontoxic biochemical etc.
- Standardized electrodes on paper substrates can be further used to carry out various processes of electrically sensitive organic molecules and analytes in miniaturized settings.
- The utility of the proposed device can also be extended to develop standardized electrodes with wax, correction pens or other similar materials for diverse point-of-care applications.

## Methods and materials

### Chemicals and material

All chemicals were purchased from Alfa Aesar, India. Different pore sizes of Whatman filter paper Grades such as Gr 1, 6 were purchased from Sigma Aldrich, India. Multiple grades (HB, 8B) of Graphite pencils (Apsara brands) were purchased from a local stationery store. Graphite pencils (HB, 8B) were used for electrodes fabrication by automated pencil stroke device with uniformity, and constant force on porous nature filter paper. A PLA (polylactic acid) filament (FibReel, 1.75 mm, Rever Ind. procured from Sigma Aldrich, India) was used to fabricate the automatic pencil stroke device and its components by FDM based dual extruder 3D printer (Creator Pro, USA). The Force Sensing Resistor (FSR) sensor was used to identify the force (N) at the time of graphite electrode fabrication. The FSR sensor (Interlink 402 model, 0.5″ sensing diameter) was procured from Interlink Electronics, India. Graphtec CE 6000-60 cutting plotter (X–Y motion) was used to fabricate graphite electrodes. Graphtec CE6000-60 cutting plotter was purchased from Graphtec America Inc. CE 6000 series, US.

### Automated pencil stroke device fabrication/assembly

**Figure 5:**
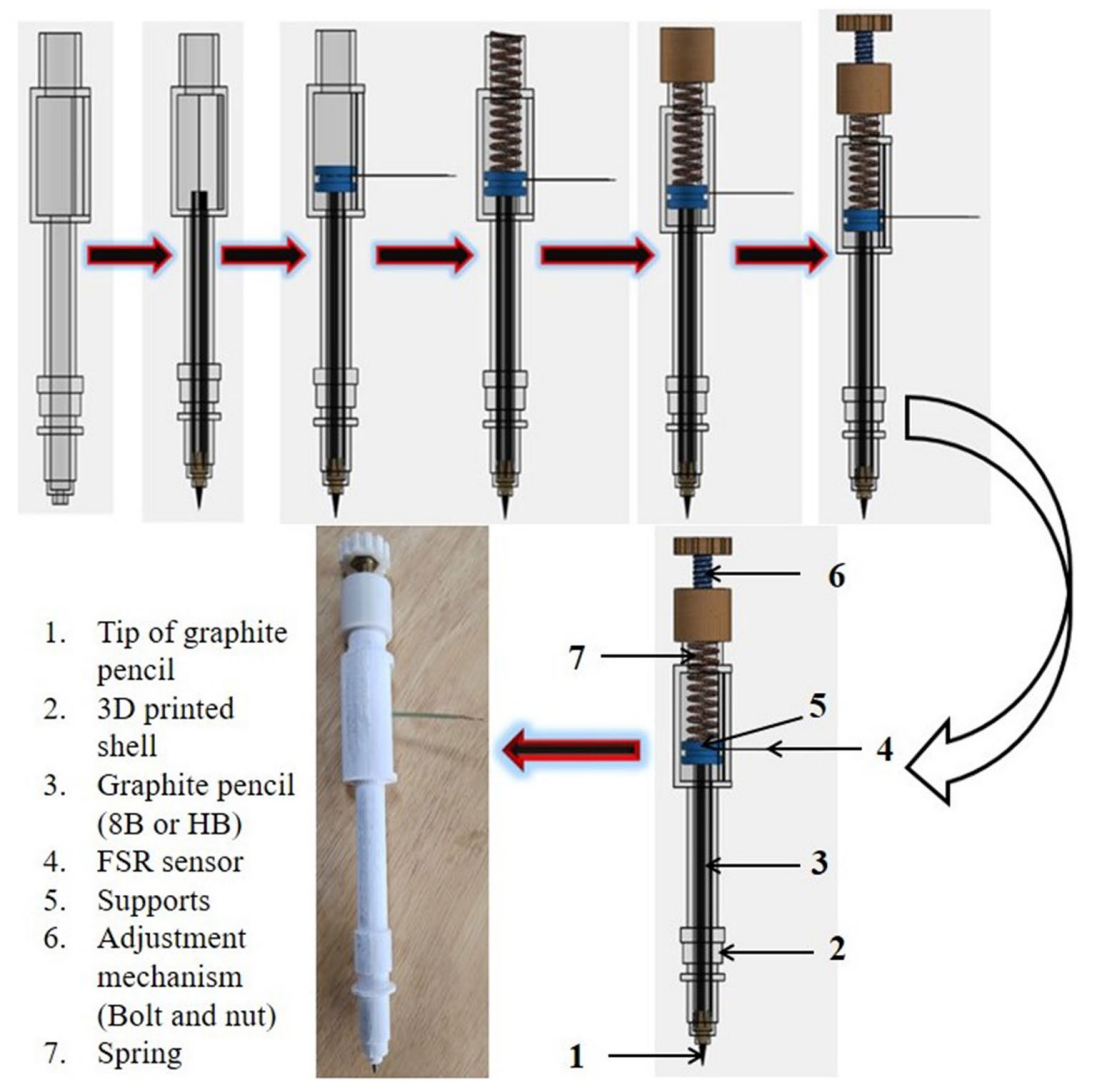
Detailed step-by-step fabrication procedure and a prototype model of automated pencil stroke device with FSR sensor.

Prior to the automated pencil stroke device fabrication, FDM based 3D printer was used to fabricate an automated pencil stroke device and its components with selected dimensions. In the beginning, virtual design of an automated pencil stroke device, with various components, was created with the help of SolidWorks 2013 modeling software. Detailed step-by-step fabrication procedure and prototype of an automated pencil stroke device is mentioned in Fig. 5. The device assembly and disassembly videos are available in the supplementary as Video S2 and S3, respectively. Further, Table 2 shows a thorough description of the components of the automated pencil stroke device and their technical features with functions. Exhaustive dimensions of the automated pencil stroke device and assembly are given in Fig. S3. An FSR sensor was used to observe the force (N) at the time of graphite electrode fabrication on porous cellulose paper (Whatman filter paper). The schematic illustration and circuit diagram of the FSR sensor are shown in Fig. S1^22^.

**Table 2:** Detail description of an automated pencil holder device components.

S. no.	Component/software	Technical feature	Function/role
1	SolidWorks 2013 × 64 version	3D Modelling Software	To create 3D models for printing
2	AutoCAD 2020	2D Drawing Software	To create an electrode configuration for making
3	Flash print	Convert ‘.stl.’ file to a 3D Printer compatible format	Used to assign the type of filament used for parts design, their melting point
4	Flash Forge Creator Pro 3D Printer	Dual extruder printer with 1.75 mm filament compatibility	To print the designs based on the fed file
5	Unique Laser System (VLS Series)	CO_2_ Laser capable of cutting and engraving	To cut the paper with precision
6	Microcontroller/Arduino Uno	Easily programmable and compact	Acts as the control unit, integrates all electronic components
7	Graphtec Cutting Plotter (CE6000-60 series)	Liner movement	For electrode making
8	FSR Sensor (402 series)	Force detector	To identifying the force on electrodes
9	Graphite pencils (different grades)	Carbon nature pencils	For graphite electrodes materials
10	Whatman cellulose filter paper	Porous nature filter paper	To create graphite electrodes on cellulose filter papers

As can be seen, a spiral spring, with bolt and nut mechanism, was used to adjust the force on the FSR sensor and graphite electrodes fabrication time to maintain constant force and uniformity of graphite particles deposition on the porous cellulose paper (Whatman Gr 1 filter paper).

### Automated pencil stroke device setup with plotter

**Figure 6:**
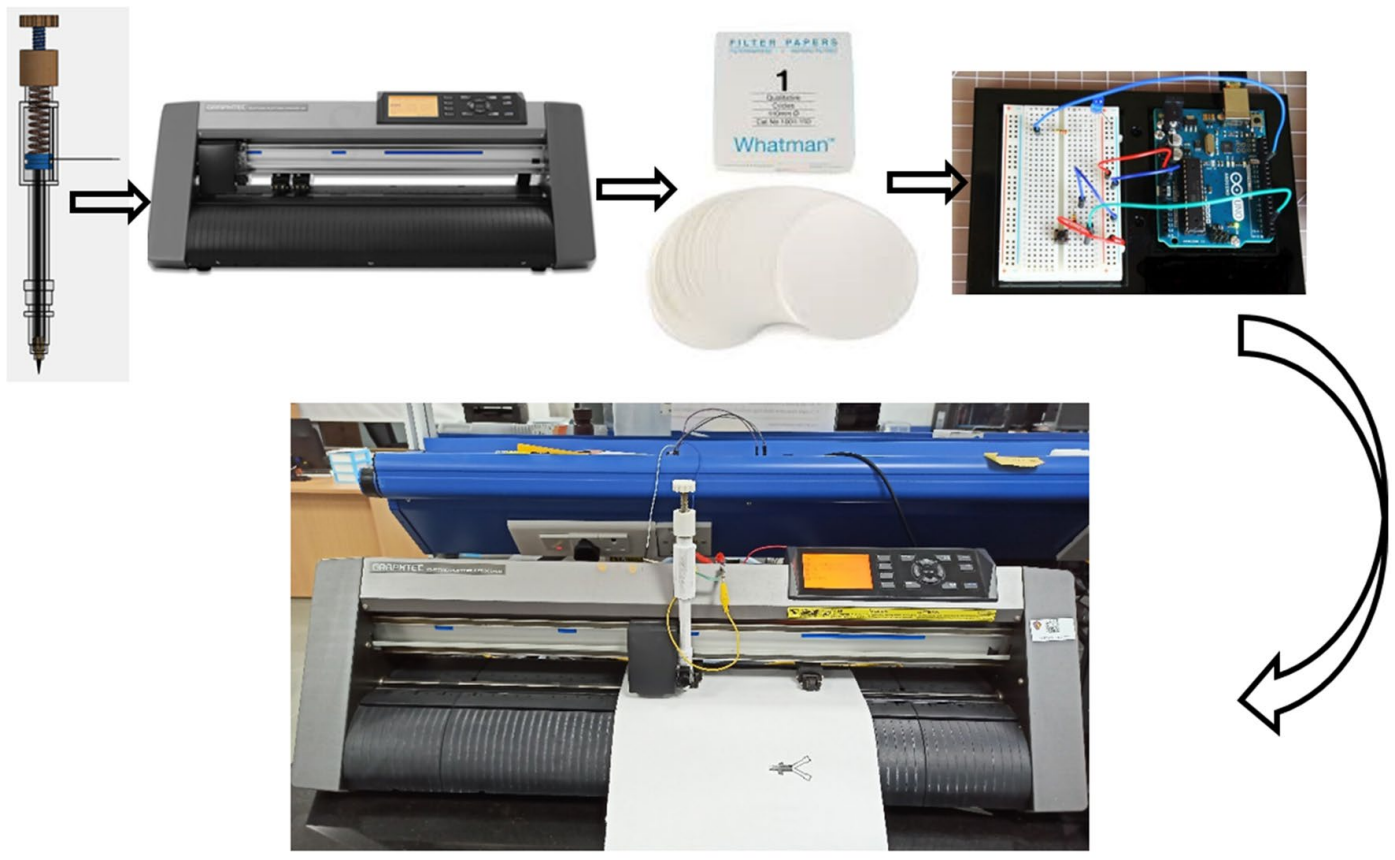
Complete graphite electrodes fabrication setup of an automated pencil stroke device with a plotter.

Graphtec plotter was used to hold the pencil stroke device for electrodes fabrication with varies multiple strokes and to draw the pencil strokes on a porous paper based substrate. The complete experimentation setup of an automated pencil stroke device with plotter is illustrated in Fig. 6.

**Figure 7:**
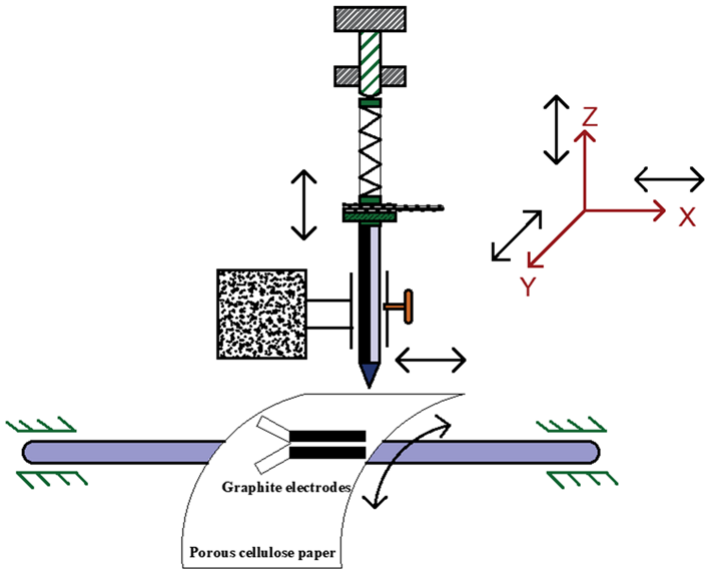
A complete representation of automated graphite pencil holder orientation (motion) with graphtec plotter.

The complete schematic representation of graphite pencil holder with the motions (XYZ orientations) mechanism of the plotter is depicted in Fig. 7. Here, porous cellulose paper revolves around Y-axis (CW and CCW direction) for creating pencil graphite zones (as electrodes) on it. Moreover, the graphite pencil holder device moves around X-axis (left to right) for multiple strokes, and the automated device moves in Z-axis (top and bottom) for sensing the force magnitude at the time to realize the pencil graphite zones.

**Figure 8:**
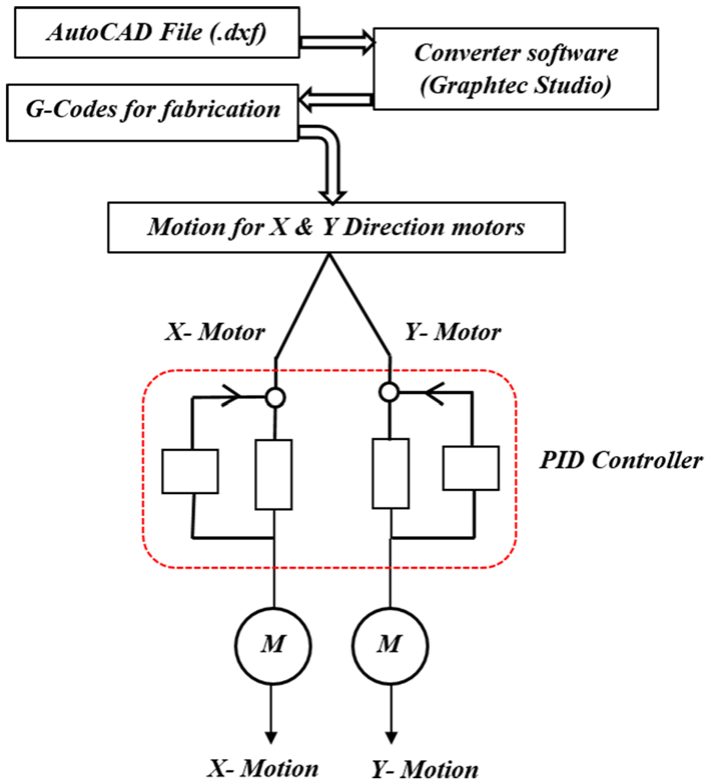
The outline representation of graphite electrodes fabrication with graphtec plotter motions.

Here, two technical supporting softwares were employed for drawing graphite pencil strokes: Graphtec studio (GS) for operating the Graphtec plotter and AutoCAD to design the electrodes configuration. After designing the geometry of the electrodes, it has been converted into .dxf file and imported into Graphtec studio (GS) for automated pencil strokes. The complete outline representation of graphite electrodes fabrication with graphtec plotter motions (X, Y), graphite electrodes design procedure is mentioned in Fig. 8. Here, prior to the designing of the electrodes by AutoCAD software, that file was saved in .dxf format and convert into G-codes for X, Y motions (graphtec plotter) by graphtec studio software. All technical functions of automated pencil holder with graphtec plotter are summarized in Table 2. Moreover, the number of strokes can also be varied by the Graphtec plotter.

Evidently, most of the electrodes, on a paper microfluidic device, are fabricated manually and therefore are without uniformity in the quantity of force applied on electrodes. To overcome this, an FSR passive sensor was used for identifying the amount of force applied on electrodes at a particular fabrication time. For the FSR sensor, a microcontroller was used to operate a sensor for identifying the applied force on electrodes through MATLAB coding. This microcontroller was connected to the PC to communicate the force data. Afterwards, the Arduino Uno app was used to execute a Matlab program for capture, analyse, and tune the Force data.

## Supplementary information

Supplementary Information 1.

Supplementary Video S1.

Supplementary Video S2.

Supplementary Video S3.

## References

[1] Goel S (2018). From waste to watts in micro-devices: review on development of membraned and membraneless microfluidic microbial fuel cell. Appl. Mater. Today.

[2] Kjeang E, Djilali N, Sinton D (2009). Microfluidic fuel cells: a review. J. Power Sources.

[3] Wang ZL, Wu W (2012). Nanotechnology-enabled energy harvesting for self-powered micro-/nanosystems. Angew. Chem. Int. Ed..

[4] Annu P, Sharma S, Jain R, Raja AN (2020). Review—pencil graphite electrode: an emerging sensing material. J. Electrochem. Soc..

[5] David IG, Popa DE, Buleandra M (2017). Pencil graphite electrodes: a versatile tool in electroanalysis. J. Anal. Methods Chem..

[6] Aoki K, Okamoto T, Kaneko H, Nozaki K, Negishi A (1989). Applicability of graphite reinforcement carbon used as the lead of a mechanical pencil to voltammetric electrodes. J. Electroanal. Chem..

[7] Bandapati M, Krishnamurthy B, Goel S (2019). Fully assembled membraneless glucose biofuel cell with MWCNT modified pencil graphite leads as novel bioelectrodes. IEEE Trans. Nanobioscience.

[8] Bandapati M, Rewatkar P, Krishnamurthy B, Goel S (2019). Functionalized and enhanced HB pencil graphite as bioanode for glucose-O_2_ biofuel cell. IEEE Sens. J..

[9] Rewatkar P, Bandapati M, Goel S (2019). Miniaturized additively manufactured co-laminar microfluidic glucose biofuel cell with optimized grade pencil bioelectrodes. Int. J. Hydrogen Energy..

[10] Arun RK, Gupta V, Singh P, Biswas G, Chanda N (2019). Selection of graphite pencil grades for the design of suitable electrodes for stacking multiple single-inlet paper-pencil fuel cells. ChemSelect.

[11] Arun RK, Halder S, Chanda N, Chakraborty S (2014). A paper based self-pumping and self-breathing fuel cell using pencil stroked graphite electrodes. Lab Chip..

[12] Veerubhotla R, Bandopadhyay A, Das D, Chakraborty S (2015). Instant power generation from an air-breathing paper and pencil based bacterial bio-fuel cell. Lab Chip..

[13] Ye D (2013). Performance of a microfluidic microbial fuel cell based on graphite electrodes. Int. J. Hydrogen Energy.

[14] Lal S, Janardhanan VM, Deepa M, Sagar A, Sahu KC (2015). Low cost environmentally benign porous paper based fuel cells for micro-nano systems. J. Electrochem. Soc..

[15] Shen LL, Zhang GR, Venter T, Biesalski M, Etzold BJM (2019). Towards best practices for improving paper-based microfluidic fuel cells. Electrochim. Acta.

[16] Eikerling M, Kornyshev AA, Kuznetsov AM, Ulstrup J, Walbran S (2002). Mechanisms of proton conductance in polymer electrolyte membranes. J. Phys. Chem. B..

[17] Rao LT, Dubey SK, Javed A, Goel S (2020). Statistical performance analysis and robust design of paper microfluidic membraneless fuel cell with pencil graphite electrodes. J. Electrochem. Energy Convers. Storage.

[18] Rao LT, Rewatkar P, Dubey SK, Javed A, Goel S (2020). Performance optimization of microfluidic paper fuel-cell with varying cellulose fiber papers as absorbent pad. Int. J. Energy Res..

[19] Jung DG, Ahn Y (2020). Microfabricated paper-based vanadium co-laminar flow fuel cell. J. Power Sources.

[20] Lehmann M, Eisengräber-Pabst J, Ohser J, Moghiseh A (2013). Characterization of the formation of filter paper using the Bartlett spectrum of the fiber structure. Image Anal. Stereol..

[21] Sousa MC, Buchanan JW (2000). Observational models of graphite pencil materials. Comput. Graph. Forum.

[22] The Interlink FSR 402 sensor datasheet and technical specification, web source https://www.trossenrobotics.com/productdocs/2010-10-26-DataSheet-FSR402-Layout2.pdf.

