# Supplementary material for: Automated pencil electrode formation platform to realize uniform and reproducible graphite electrodes on paper for microfluidic fuel cells
Source: Sci Rep. 2020 Jul 15;10:11675. doi: 10.1038/s41598-020-68579-x (PMC7363794; doi:10.1038/s41598-020-68579-x)
Supplement: Supplementary file 1 — Supplementary Information 1. [file 41598_2020_68579_MOESM1_ESM.docx]

Supplementary Data

**Automated Pencil Electrode Formation Platform to Realize Uniform and Reproducible Graphite Electrodes on Paper for Microfluidic Fuel Cells**

Lanka Tata Rao^1^, Prakash Rewatkar^2^, Satish Kumar Dubey^1^, Arshad Javed^1^, and Sanket Goel^2*^

^1^Department of Mechanical Engineering, Birla Institute of Technology and Science (BITS) Pilani, Hyderabad Campus, Hyderabad, India 500078

^2^MEMS, Microfluidics and Nanoelectronics Lab, Department of Electrical and Electronics Engineering, Birla Institute of Technology and Science (BITS) Pilani, Hyderabad Campus, Hyderabad, India 500078

*E-mail: [sgoel@hyderabad.bits-pilani.ac.in](mailto:sgoel@hyderabad.bits-pilani.ac.in) and [sanketgoel@gmail.com](mailto:sanketgoel@gmail.com)

| 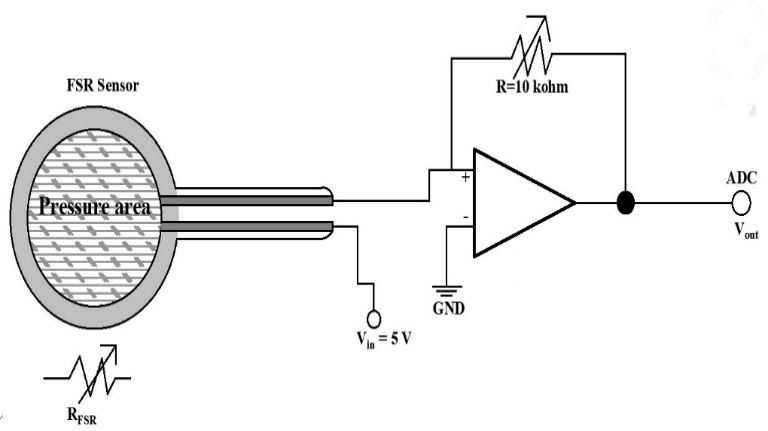  **(a)** | 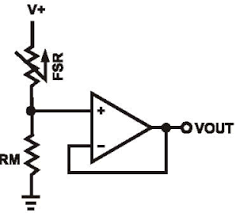  **(b)** |
| --- | --- |
| 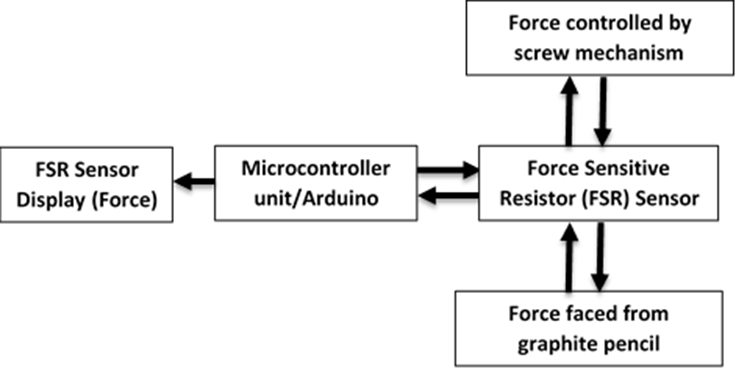  **(c)** | |
| Fig. S1. FSR sensor, (a ) Block diagram, (b) Circuit diagram, and (c) Step by step procedure of FSR sensor operation. | |

| 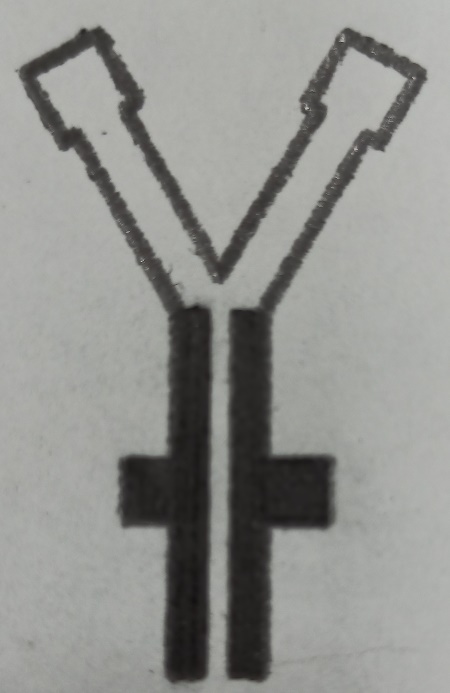  **(a)** | 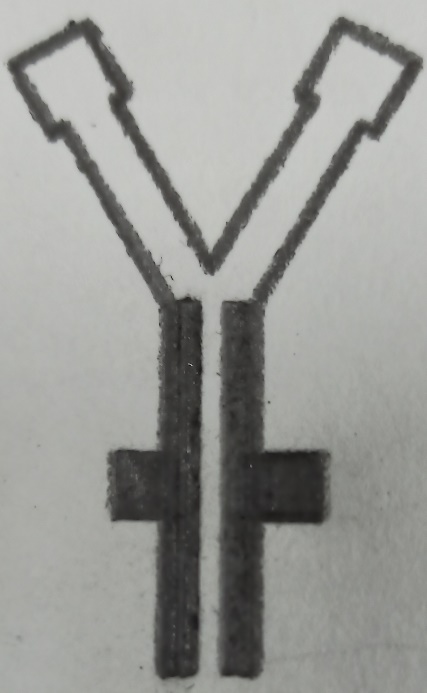  **(b)** | 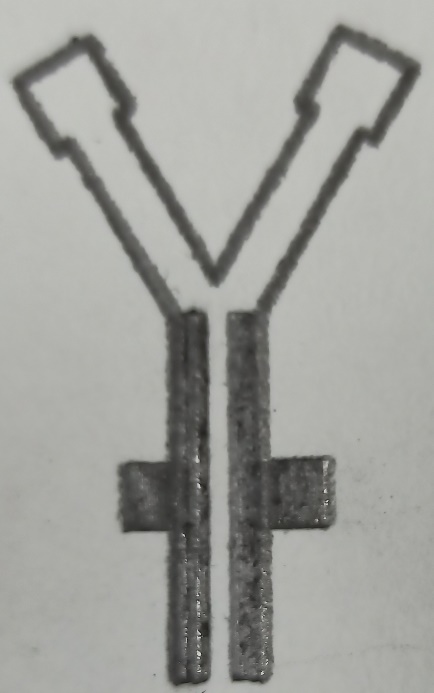  **(c)** |
| --- | --- | --- |
| 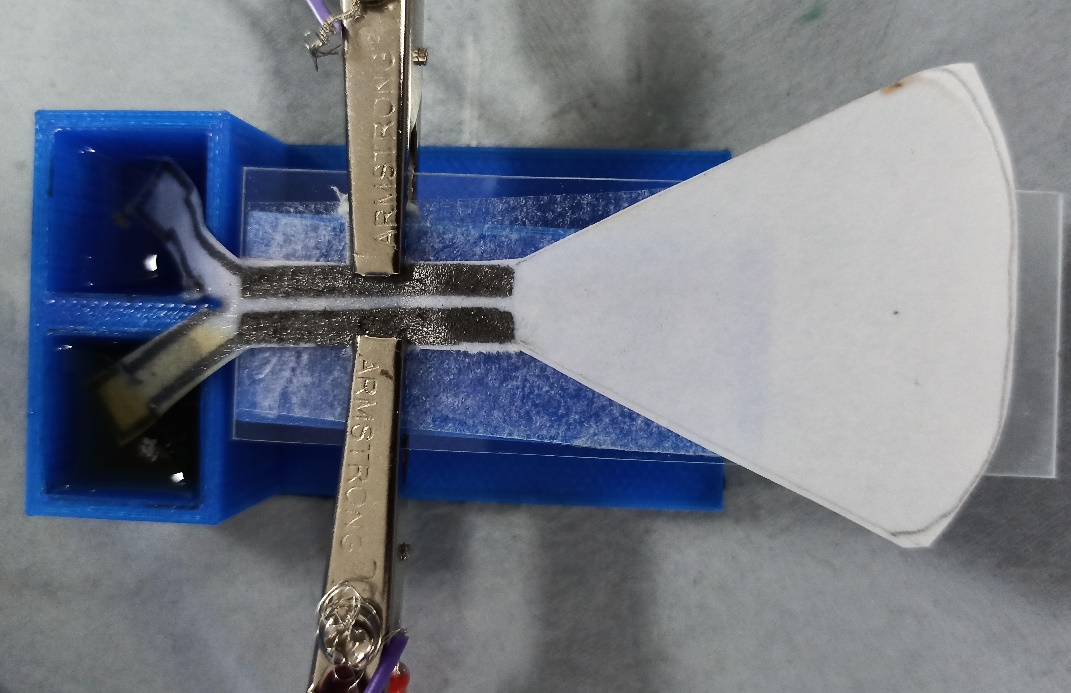  **Formic acid (1 M)**  **Sulphuric acid (3.75 M)**  **8B graphite electrode**  **HB graphite electrode**  **Absorbent pad**  **(d)** | | |
| Fig.S2. the microfluidic fuel cell with automated graphite electrodes, (a) 100 stroke, (b) 50 stroke, (c) 30 stroke electrodes, and (d) Experimentation setup with 100 stroke electrodes | | |

| **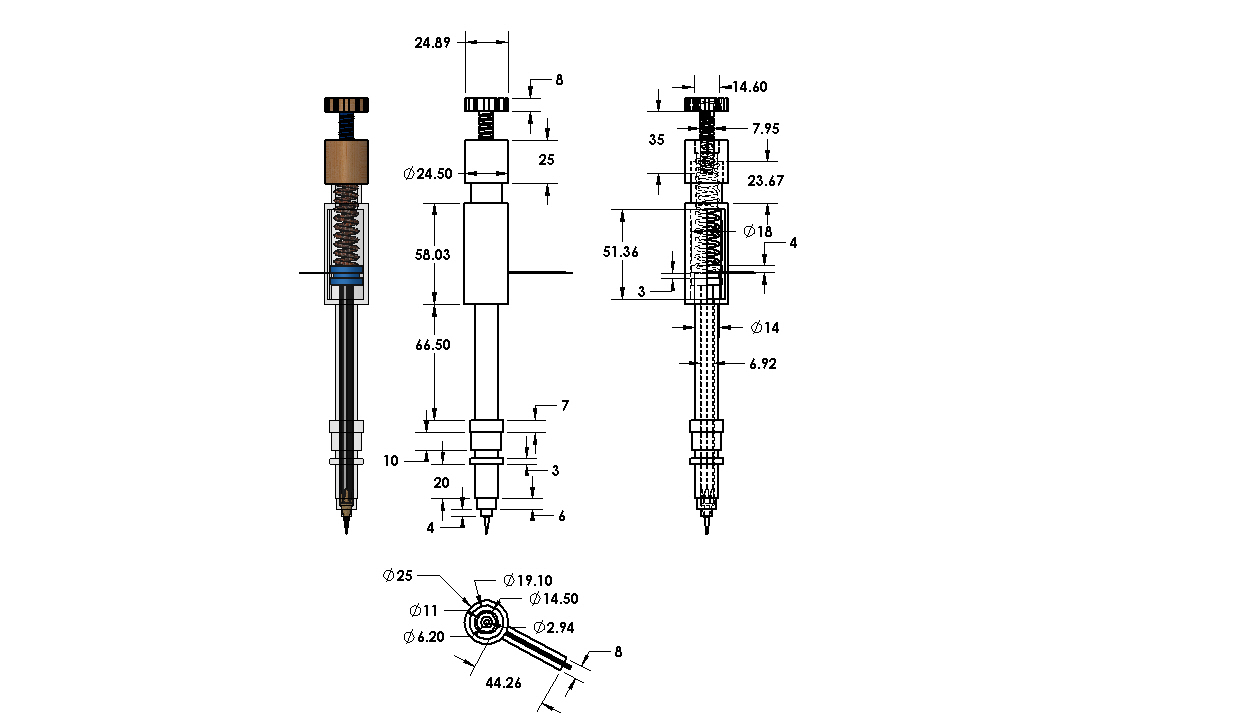**  **(a)** |
| --- |
| 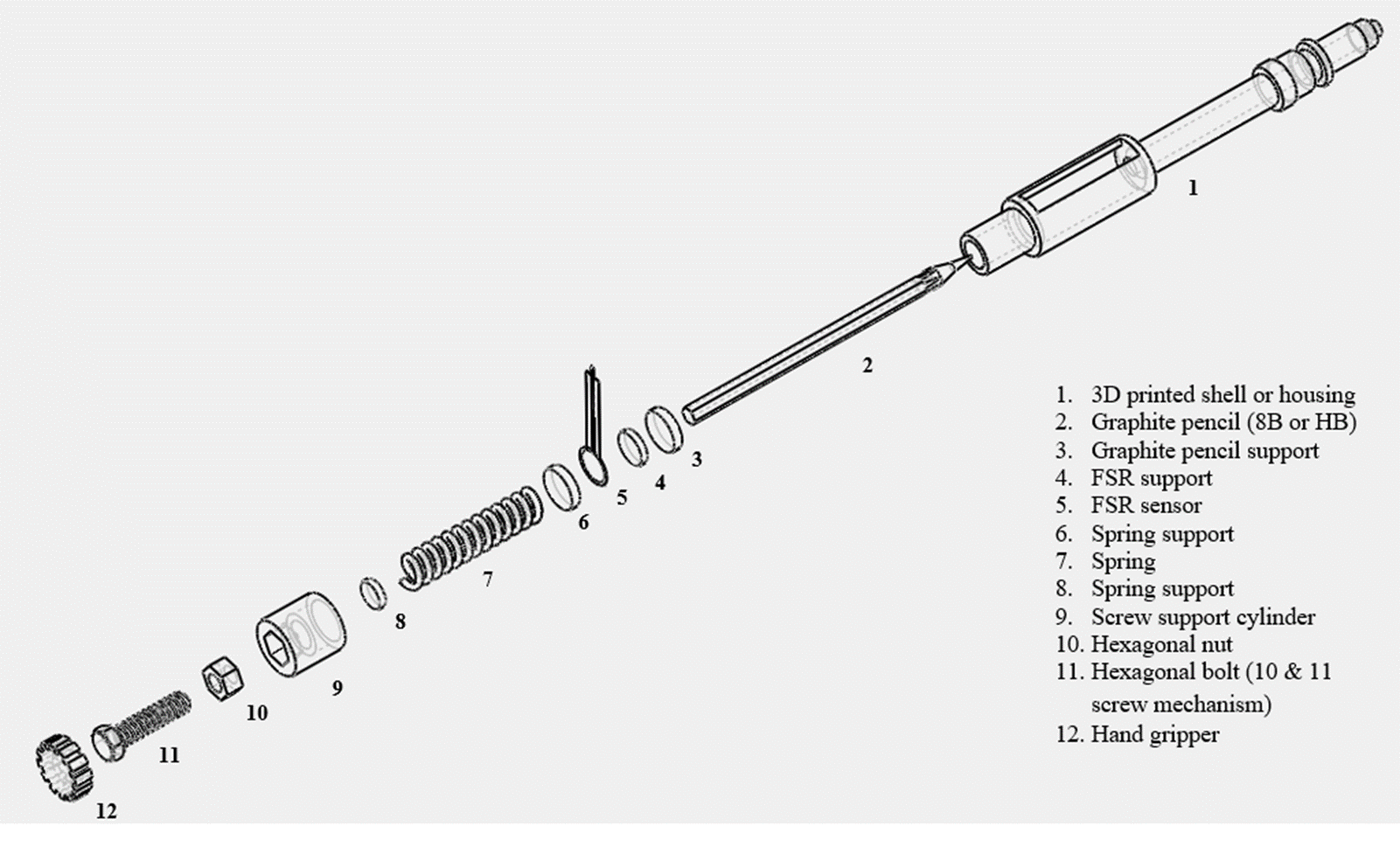  **(b)** |
| 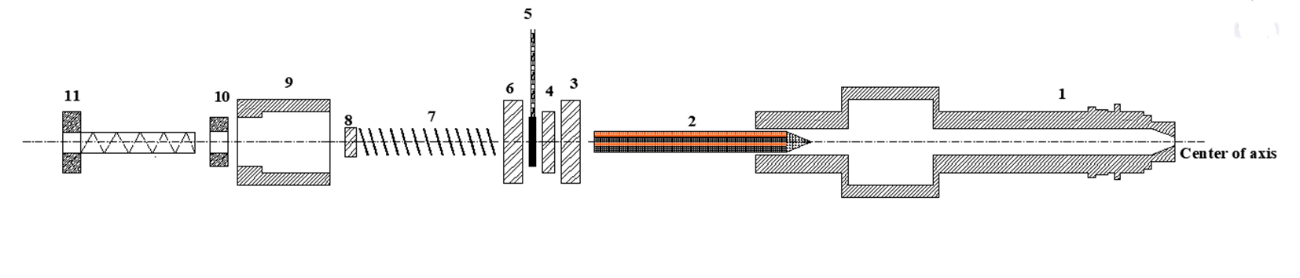  **(c)**   1. 3D printed shell or housing 2. Graphite pencil (8B or HB) 3. Graphite pencil support 4. FSR support 5. FSR sensor 6. Spring support 7. Spring 8. Spring support 9. Screw support cylinder 10. Hexagonal nut 11. Hexagonal bolt (10 & 11 screw mechanism) |
| Fig. S3. (a) Detail dimensions of an automated pencil stroke device with FSR sensor, (b) automated pencil stroke device assembly procedure, (c) Exploded view of automated pencil stroke device |
